# Malpractice Liability and Defensive Medicine: A National Survey of Neurosurgeons

**DOI:** 10.1371/journal.pone.0039237

**Published:** 2012-06-22

**Authors:** Brian V. Nahed, Maya A. Babu, Timothy R. Smith, Robert F. Heary

**Affiliations:** 1 Department of Neurosurgery, Massachusetts General Hospital, Boston, Massachusets, United States of America; 2 Department of Neurological Surgery, Mayo Clinic, Rochester, Minnesota, United States of America; 3 Department of Neurological Surgery, Northwestern University, Chicago, Illinois, United States of America; 4 Department of Neurological Surgery, University of Medicine and Dentistry of New Jersey, Newark, New Jersey, United States of America; University of Louisville, United States of America

## Abstract

**Background:**

Concern over rising healthcare expenditures has led to increased scrutiny of medical practices. As medical liability and malpractice risk rise to crisis levels, the medical-legal environment has contributed to the practice of defensive medicine as practitioners attempt to mitigate liability risk. High-risk specialties, such as neurosurgery, are particularly affected and neurosurgeons have altered their practices to lessen medical-legal risk. We present the first national survey of American neurosurgeons’ perceptions of malpractice liability and defensive medicine practices.

**Methods:**

A validated, 51-question online-survey was sent to 3344 practicing U.S. neurosurgeon members of the American Association of Neurological Surgeons, which represents 76% of neurosurgeons in academic and private practices.

**Results:**

A total of 1028 surveys were completed (31% response rate) by neurosurgeons representing diverse sub-specialty practices. Respondents engaged in defensive medicine practices by ordering additional imaging studies (72%), laboratory tests (67%), referring patients to consultants (66%), or prescribing medications (40%). Malpractice premiums were considered a “major or extreme” burden by 64% of respondents which resulted in 45% of respondents eliminating high-risk procedures from their practice due to liability concerns.

**Conclusions:**

Concerns and perceptions about medical liability lead practitioners to practice defensive medicine. As a result, diagnostic testing, consultations and imaging studies are ordered to satisfy a perceived legal risk, resulting in higher healthcare expenditures. To minimize malpractice risk, some neurosurgeons have eliminated high-risk procedures. Left unchecked, concerns over medical liability will further defensive medicine practices, limit patient access to care, and increase the cost of healthcare delivery in the United States.

## Introduction

Over the past three decades, medical malpractice premiums have risen disproportionately compared to physician incomes. Between 2000 and 2002, there was a 15% rise in the cost of medical malpractice insurance while physician incomes decreased during this time [1]. Although malpractice litigation facilitates recourse against physician negligence, it also creates opportunities for frivolous lawsuits. Malpractice crises have affected many parts of the country over the past several decades, influencing the locations in which physicians practice, the types of procedures offered, and ultimately, access to healthcare. Physicians practice defensive medicine due to concern for liability risk, which contributes to an increase in healthcare expenditures. As these are politically sensitive topics, recent efforts to reform the healthcare system have largely ignored medical malpractice and tort reform. This paper is the first national survey specifically aimed at investigating the impact of liability risk perception on access to healthcare.

### History

Medical malpractice was uncommon in the United States until the 19^th^ century [2]. Malpractice litigation emerged, in part, in response to the declining role of religion as justification for personal injury and a rising sense that physical well-being could be controlled and even improved [3]–[4]. As the lay press reported medical advances, patients shifted their beliefs towards a feeling that diseases were treatable. Poor outcomes were scrutinized as to whether a physician could have or should have performed better [3]. Between 1840 and 1860, the number of malpractice cases carried to state appellate courts in the United States increased over 950% and malpractice litigation jumped roughly 10-fold compared to population growth [5]. Medical journals published the first papers on malpractice around this time [6].

Three major reasons account for the increased numbers of malpractice cases since 1840. First, while medical advances improved healthcare overall, unintended side effects of treatments became fertile ground for litigation. For example, radiographs improved diagnostic abilities but the first patients were exposed to excessive radiation doses or improper interpretation of images [7]. Second, as organizations developed uniform standards of training, licensing, and practicing, doctors could be held accountable for deviating from prescribed norms [8]–[9]. Finally, while the advent of malpractice insurance for physicians protected personal assets, it led to the establishment of malpractice litigation as a recognized legal instrument.

**Table 1:** Demographic Information.

1 Clinical Status		
<5 years in practice		12.6%
5–10 years in practice		15.3%
10–20 years in practice		36.3%
20–30 years in practice		25.0%
>30 years in practice		10.6%
2 Gender		
Male		91.8%
Female		8.1%
3 Number of Annual Operative Cases		
<50		1.0%
50–100		4.7%
100–200		24.0%
200–300		38.6%
300–400		21.0%
400–500		7.0%
>500		3.1%
4 Number of Lifetime Operative Case		
<500		1.9%
501–1000		3.7%
1001–2000		11.3%
2001–3000		17.3%
3001–4000		15.7%
4001–5000		12.9%
5001–10,000		29.9%
>10,000		7.0%
5 Type of Practice		
Academic		23.5%
Private Practice		30.4%
Mixed (Academic and Private)		13.4%
Military		0.9%
Hospital-based		13.5%
Group Practice		18.0%
Solo Practice		6.1%
6 Number of Neurosurgeons in Practice		
Solo Practioner	14.6%	
1–2	16.5%	
3–5	24.2%	
6–10	20.4%	
11–15	11.0%	
>15	13.1%	

### High-Risk Specialties

Malpractice liability affects all medical practitioners. Several studies, however, have identified specific specialties that are at “high-risk” for litigation including: Emergency Medicine, General Surgery, Orthopedic Surgery, Neurosurgery, Obstetrics/Gynecology, and Radiology [10]. These specialties frequently address acute medical problems that require rapid decision-making such that a poor outcome may be unavoidable. These specialties are also predominantly procedure driven and outcomes may be scrutinized for the skill of the treating physician. Physicians who cover trauma or emergencies have increased liability secondary to the increased risk of poorer outcomes in these settings compared with elective practices [11].

The acute decision-making required to care for ill patients, the small margin for error, and the potential for adverse outcomes are some of the reasons neurosurgery is considered a “high-risk” specialty. As malpractice liability continues to be of concern, neurosurgeons have reduced practice offerings to mitigate liability exposure. In Pennsylvania, high liability premiums and large lawsuit settlements have led some neurosurgeons to avoid intracranial operations, instead performing only elective spine operations which may have less risk of a poor outcome. Fears over malpractice have helped drive neurosurgeons from the state, and created what the Chester County Medical Society declared as a medical malpractice “crisis,” which “clearly jeopardize(s) [residents and creates] a healthcare situation of extreme concern” for neurotrauma patients who could not “receive immediate, life-saving interventions due to the lack of any full-time neurosurgeon in the county.” As a result, acutely injured patients had to be transferred to facilities with neurosurgeons; in two instances, patients died during the one-hour ambulance ride from Chester County to Lancaster County, Pennsylvania [12].

**Table 2:** Patients Served by Neurosurgeon Respondents.

1 Percentage of Patients Treated who Carry Commercial Insurance	
<10%	2.6%
10–25%	16.9%
25–50%	37.8%
50–75%	27.3%
>75%	8.0%
Not known	7.3%
2 Percentage of Patients Treated who are on Medicare	
<10%	7.5%
10–25%	29.7%
25–50%	46.0%
50–75%	9.3%
>75%	0.6%
Not known	6.6%
3 Percentage of Patients Treated who are on Medicaid	
<10%	43.8%
10–25%	34.5%
25–50%	10.2%
50–75%	2.9%
>75%	0.5%
Not known	7.9%
4 Percentage of Patients Treated who are on TRICARE	
<10%	59.5%
10–25%	8.3%
25–50%	0.8%
50–75%	0.1%
>75%	0.9%
Not known	7.9%

**Table 3:** Malpractice Premiums.

1 Malpractice Premium as a Percentage of Gross Annual Revenue	
>60%	1.2%
50–59%	1.6%
40–49%	3.2%
30–39%	6.7%
20–29%	16.6%
10–19%	39.8%
<10%	30.6%
2 Changes to Liability Premiums over the Past Three Years	
Increased >25%	8.8%
Increased >10%	21.7%
No significant change	38.3%
Decreased >10%	7.7%
Decreased >25%	1.1%
Not known	22.0%
3 Over the Past Three Years, Average Reimbursement/CPT Code Change	
Increased >25%	2.1%
Increased >10%	1.0%
No significant change	13.7%
Decreased >10%	55.8%
Decreased >25%	14.3%
Not known	14.8%
4 Number of Claims Against Survey Respondents in Past Three Years	
Zero	60.7%
1–2	34.0%
3–4	3.7%
5–6	0.8%
7–8	0.2%
9–12	0.0%
13–15	0.2%
>15	0.2%
5 Number of Settlements Made Against Respondents over their Lifetime	
1–3	36.5%
4–7	4.5%
>7	0.4%
None	58.0%

**Table 4:** Perceptions of Neurosurgeon Respondents.

1 "There is a medical liability crisis in my area"	
Strongly Agree	38.6%
Agree	34.7%
Neutral	17.4%
Disagree	7.0%
Strongly Disagree	2.1%
2 "Medical liability affects my decision on where, geographically, to practice neurosurgery."	
Strongly Agree	39.1%
Agree	32.2%
Neutral	17.0%
Disagree	9.0%
Strongly Disagree	2.5%
3 "Medical liability affects my decision on how long to continue to practice neurosurgery"	
Strongly Agree	40.0%
Agree	31.2%
Neutral	16.8%
Disagree	8.8%
Strongly Disagree	3.0%
4 "I view every patient as a potential lawsuit."	
Strongly Agree	32.0%
Agree	37.3%
Neutral	12.3%
Disagree	12.6%
Strongly Disagree	5.7%

In the field of obstetrics, concern for malpractice liability has led to changes in healthcare delivery. The Healthcare Cost and Utilization Project-Nationwide Inpatient Sample found that states in which malpractice premiums exceeded $100,000 were associated with higher incidences of cesarean deliveries (odds ratio 1.17) and lower incidences of vaginal births after cesarean deliveries (odds ratio 0.60). There were also lower rates of instrumental deliveries (odds ratio 0.72) compared with states where the average malpractice premium was less than $50,000 [13].

While the healthcare debate has focused on efforts to reduce unnecessary costs and encourage physicians to adhere to evidence-based medicine, little attention has been paid to the role of defensive medicine in exacerbating the liability crisis. This paper studies the beliefs and self-reported practices of neurosurgeons to determine how the perception of malpractice risk affects routine practice. We report the first national survey of neurosurgeons on this topic.

**Table 5:** Defensive Medicine Responses.

1 Defensive Medicine Practices done SOLELY to Minimize Risk of a Lawsuit		
Ordered lab tests	66.7%	
Referred patients	66.0%	
Prescribed mediciation	40.0%	
Suggested a procedure	36.0%	
Ordered imaging	72.0%	
2 How often do Survey Respondents Order Additional Laboratory Tests for Defensive Purposes?		
Always	9.7%	
Very Often	31.5%	
Sometimes	39.8%	
Rarely	15.4%	
Never	3.4%	
3 How often do Survey Respondents Order Additional Imaging for Defensive Purposes?		
Always		13.0%
Very Often		43.7%
Sometimes		31.7%
Rarely		9.4%
Never		2.2%
4 How often do Survey Respondents Obtain Initial Consultations for Defensive Purposes?		
Always	8.5%	
Very Often	32.2%	
Sometimes	38.0%	
Rarely	17.5%	
Never	3.5%	
5 How often do Survey Respondents Make Referrals for Defensive Purposes?		
Always	6.2%	
Very Often	26.7%	
Sometimes	42.2%	
Rarely	20.7%	
Never	4.2%	
6 What Prompted Discontinuation of High-Risk Services?		
Liability	49.7%	
Technical Skill Involves	9.2%	
Dislike	13.8%	
Changing Practice	24.9%	
Other	2.3%	
7 What are your Feelings on Malpractice Premiums Related to Maintaining Cranial Privileges		
Very Concerned	29.9%	
Somewhat Concerned	32.5%	
Neutral	19.2%	
Somewhat Unconcerned	5.1%	
Completely Unconcerned	6.1%	
Cranial Priveleges Not Maintained	1.7%	
Unknown	5.1%	
8 Greatest Concern with Maintaining Trauma Privileges		
Reimbursement	44.6%	
Malpractice	44.0%	
Unknown	10.9%	
9 Overall Burden of Liability Insurance		
Not a Burden	9.5%	
Minor Burden	26.6%	
Major Burden	49.6%	
Extreme Burden	14.3%	

## Methods

A 51-question survey comprised of previously validated questions [10], [14] was developed. This survey included questions on eight basic domains thought to influence defensive practices: 1) surgeon demographics 2) patient demographics 3) physician practice type 4) payment source: private insurance, Medicaid, Medicare, or TRICARE; 5) type of malpractice insurance carried; 6) changes to insurance premium rates or coverage types; 7) practitioner perceptions related to liability, and 8) practitioner behaviors in terms of ordering of laboratory tests and imaging studies. In a preliminary assessment, the survey was administered to a small group of 20 neurosurgical practitioners, and took 10 minutes on average to complete. The survey was then sent to all 3344 United States members of the American Association of Neurosurgeons (AANS) with a valid email address. The AANS is the largest neurosurgical society in the United States and represents 76% of neurosurgeons in academic and private practices. The survey respondents consisted of neurosurgeons in different practice settings, including: active practice; active provisional military practice; active military practice; and active provisional members. The survey was presented to the AANS members via an online survey tool and was conducted over a 6-week period. Approval from IRB and informed consent was not obtained given that this was a de-identified anonymous online survey. The purpose of this study was disclosed to the participants prior to beginning the survey.

## Results

Of the 3344 practicing neurosurgeons registered in the AANS directory, 1028 completed the questionnaire (31% response rate). All surveys are included in the subsequent analysis. Neurosurgeons from every state in the United States except for West Virginia (n = 31, 0.9% of total AANS members) participated in this survey. The types of practices in which survey respondents practice includes private practice (30%), academics (24%), group practice (18%), hospital-based practice (14%), and “mixed practices” representing both academic and private practice (13%). The practice sizes range from solo practice (15%) to practices with greater than 15 neurosurgeons (13%).

The complete results are presented in table form. Table 1 outlines demographic information and the neurosurgical experience of respondents (gender, work status, size of practice, specialties treated, and operative case experience). Table 2 outlines the types of patients seen and demonstrates the wide impact survey respondents have on meeting the neurosurgical needs of the public. Table 3 displays information related to malpractice premiums in the context of changes to payments and malpractice premiums as a percentage of physician income. Table 4 presents the results of perception questions asked to survey respondents to determine beliefs about malpractice. Table 5 outlines responses made to a series of questions related to ordering laboratory tests, imaging studies, and requesting referrals due to defensive medicine concerns. Questions regarding head trauma privileges, and the concerns associated with maintaining these, were also included.

## Discussion

This study marks the first survey identifying perceptions among neurosurgeons of malpractice liability and its impact on healthcare delivery. Malpractice liability concerns impact neurosurgical practice regardless of the type of reimbursement received or the patient population served. These concerns are widespread and affect neurosurgeons nationally from a diverse range of practice types serving varied patient populations. In our study, over 40% of survey respondents with malpractice concerns served between 25–50% Medicare and between 10–50% Medicaid funded patients.

### Scope of Concern

Practice patterns are strongly influenced by a practitioner’s perception of the medico-legal environment and potential malpractice risk. Seventy-two percent of respondents “strongly agreed” or “agreed” that there is a medical liability crisis in their practice area. Furthermore, 50% of neurosurgeons cited liability premiums as a “major burden,” and 14% labeled liability premiums as an “extreme burden.” In a recent study, 19.1% of neurosurgeons face a malpractice claim yearly [15]. Importantly, the impact of these lawsuits is far-reaching as practice behavior is influenced by news of a high-profile lawsuit elsewhere within the medical community [16]. This pattern may have broad implications. In our survey, 41% percent of neurosurgeons reported at least one legal settlement in their career. Regardless of actual outcomes, the threat of litigation influences how neurosurgeons utilize defensive medicine practices [18].

### Perception Changes Actions

While long perceived among physicians, this study is the first to capture the high proportions of practitioners who use defensive medicine in day-to-day patient management. Our survey revealed that 72% of respondents ordered imaging studies, 67% ordered laboratory tests, and 66% consulted other physicians solely for defensive purposes. Defensive practices are associated with increased healthcare expenditures. Several studies estimate that unnecessary costs incurred due to ordering imaging or laboratory tests primarily to lessen malpractice risk is between $41 billion over five years [17] and $55.6 billion in 2008.

Defensive medicine practices satisfy a theoretical legal standard over traditional medical practices; however, over time, these become the new *standard* practice. For example, patients with back pain often undergo magnetic resonance imaging (MRI) of the spine to protect for legal liability should the patient have a surgical lesion. While physicians in the past may have used a thorough history and physical to guide imaging, in this study, 72% of neurosurgeons surveyed stated that they order additional imaging studies solely to mitigate liability risk. This suggests that in reality, imaging is becoming a standard part of the initial workup.

Multiple studies have explored overuse of medical interventions, including but not limited to imaging studies and obtaining laboratory tests [19], [20]. Geographic variability in use and overuse has also captured much public attention, and the often-cited Dartmouth Atlas project, which describes inconsistencies in procedural volume across the country, has driven several legislative changes, including language within health reform legislation [21]. Recently, several areas of medical practice, most notably cardiology, have experienced significant reimbursement changes through the Centers for Medicare and Medicaid Services, driven in part by perceptions of overuse of certain procedures and imaging studies [22].

With its emphasis on controlling costs and affecting clinical practice, many had hoped that the Affordable Care Act would tackle issues of malpractice liability and defensive medicine. Instead, the Act fell short. The Act includes provisions (1) to extend federal malpractice protections to non-medical personnel working in free clinics and (2) authorizes $50 million over the next five years for the Department of Health and Human Service to award demonstration project grants to states to create and evaluate alternatives to the current tort litigation system for resolving disputes about injuries caused by physicians providing medical care [23]. While these direct provisions within the Act related to malpractice, additional provisions related to implementation of health reform, are feared to increase burdens placed upon physicians, and may work to exacerbate defensive medicine practices. As health reform is implemented, whether these perceptions will translate into reality will remain to be seen.

### Limiting Access to Care

Malpractice liability premiums, influenced by the services offered and the local malpractice environment, have an appreciable impact on the availability of neurosurgical care in the country. Of survey respondents, 71% “strongly agreed” or “agreed” that their chosen geographic location was influenced by medical malpractice liability concerns.

Over 50% of neurosurgeons surveyed have tailored their practice to minimize their risk of liability by eliminating “high-risk” procedures, such as those involved with traumatic head and spine injuries, intracranial hemorrhages, tumor resections, and hydrocephalus. As a result, 45% of respondents do not currently treat these high-risk conditions due to liability concerns. Further contributing to the declining number of neurosurgeons offering these high-risk procedures, 71% of neurosurgeons indicate that the malpractice environment affects their decision of how long to practice. The reduction in the number of neurosurgeons available to offer potentially life-saving procedures is magnified in a small specialty where even small limitations in access to care can have profound implications.

### Reform

States that have enacted tort reform measures have seen declines in the number of malpractice lawsuits filed and the resultant costs of medical malpractice [19]. Following the passage of Texas’ tort reform law, the prevalence of lawsuits filed per 100,000 procedures performed dropped from 40 to 8 lawsuits per 100,000 procedures before and after reform, respectively (p<0.01) [24]. Virtually all of the liability and defense costs were in the pre-tort reform period: $595,000/year versus $515/year in the post-reform environment (p<0.01).

Several models have been proposed to respond to the malpractice crisis. One model incorporates physician disclosure of medical errors [25]. Other models recommend *health courts*, specialized courts with judges trained in healthcare, which are meant to limit the number of frivolous lawsuits [26]. A third model implements patient indemnity insurance to protect patients proactively against personal losses incurred from medical interventions [26]. State-based limitations on non-economic damages, such as California’s Medical Injury Compensation Reform Act (MICRA), have also been used to curb increasing malpractice costs [27].

Ultimately, regardless of the malpractice models proposed, measures to protect high-risk practitioners are necessary to assure that patients have access to high-risk, potentially life-saving procedures. Without more protective measures, defensive practices will force the standard of medical care to satisfy a theoretical legal standard meant to address perceived liability risk rather than utilize medical judgment. As the liability crisis worsens, access to key neurosurgical procedures will continue to be curtailed for at risk populations who need them most.

### Limitations

There are several limitations affecting this study. First, a survey of practitioner perceptions may differ from actual practice patterns. The results presented in this study are dependent on each individual neurosurgeon’s responses, and thus, are subject to a response bias, with respondents perhaps more concerned about liability than non-responders. Second, this survey provides information on attitudes at a single point in time; a longitudinal series of surveys would provide more information as to whether practitioners’ views have changed and how self-reported behaviors may correspondingly be altered. Third, an anonymous survey may result in more extreme responses if the subject of the survey (namely, malpractice) is a source of frustration and/or anxiety. As mentioned, while surveys may be subjected to response bias, this study sought to identify individual perceptions and the effects of these perceptions on medical practices; therefore, utilizing a survey instrument is an ideal method to identify individual attitudes and defensive medicine practices.

### Conclusion

Balancing medical oversight with limitations on malpractice is important to uphold standards of high-quality medical care and ensure physicians do not make decisions solely for fear of litigation. The survey respondents indicated that malpractice liability results in defensive medicine practices to lessen malpractice exposure. Reductions in offering “high-risk” cranial procedures have decreased access to care for potentially life-saving neurosurgical procedures. With increasing malpractice premiums and decreasing provider reimbursement, neurosurgeons have adopted defensive measures to mitigate perceived liability risk. Without reform, malpractice premiums will continue to rise, and the number of lawsuits filed frivolously or intended for financial remuneration through settlement will go unchecked. Access to neurosurgeons and neurosurgical care will continue to be restricted which will adversely affect delivery and cost of healthcare in the United States.
